# Interventional Radiology Awareness Among Family Physicians and General Practitioners in Jazan Region, Saudi Arabia

**DOI:** 10.7759/cureus.68715

**Published:** 2024-09-05

**Authors:** Ali M Hendi, Raed E Jarram, Abdulmajeed A Jadah, Jalal H Majhali, Mohammed E Mojiri, Fahad Y Moafa, Yara A Jandali, Fouad I Hakami, Nouf M Hakami, Salma A Tahiri, Entisar M Humedi, Amani A Mutaen, Njoud A Al Alhadi, Almutasim A Arawi, Ali M Shawish

**Affiliations:** 1 Department of Radiology, Jazan University, Jazan, SAU; 2 College of Medicine, Jazan University, Jazan, SAU; 3 College of Nursing and Health Sciences, Jazan University, Jazan, SAU; 4 College of Applied Medical Sciences, Jazan University, Jazan, SAU

**Keywords:** awareness, cross-sectional survey, family medicine, interventional radiology, jazan, knowledge, minimally invasive procedures, saudi arabia

## Abstract

Background

Interventional radiology (IR) utilizes minimally invasive procedures guided by imaging to diagnose and treat various conditions, offering less invasive alternatives to traditional surgery. Despite its importance, awareness among family medicine practitioners can vary, affecting patient care. While IR has advanced in Saudi Arabia, there are limited data on family medicine practitioners' understanding of IR. This study assesses awareness of IR procedures among family medicine doctors in Jazan and their perceived need for further education.

Methods

A cross-sectional interview-administered survey was conducted online among family medicine doctors in Jazan via social media. The survey assessed demographic data, awareness of IR procedures, self-rated knowledge, and attitudes towards IR. Participants’ understanding of IR training, hospital privileges, outpatient clinics, and recognition by the Saudi Commission for Health Specialties (SCHS) was evaluated. Data were analyzed using descriptive statistics and chi-square tests.

Results

Out of 395 respondents, the age distribution was as follows: 20-29 years (44.3%), 30-39 years (32.9%), and 40 years or older (22.8%). Gender distribution was as follows: females (44.6%) and males (55.4%). Specialties included family medicine consultants (10.6%), residents (32.4%), specialists (22.8%), and general practitioners (34.2%). Awareness of IR procedures varied: uterine fibroid embolization (58.7%), coronary angiography (57.5%), vascular angioplasty (63.5%), radiofrequency ablation (61.3%), peripheral vascular bypass (61.8%), brain biopsy (56.2%), nephrostomy tube placement (59.5%), varicose veins treatment (63.0%), and cystoscopic tumor resection (54.7%). Self-rated knowledge was as follows: poor (46.8%), adequate (27.1%), good (15.7%), and excellent (10.4%). Most believed that interventional radiologists’ training was in radiology (62.8%), with fewer attributing it to vascular surgery (20.5%) or a combination (16.7%). Regarding privileges and facilities, 248 (62.8%) reported hospital admitting privileges for IRs, 251 (63.5%) reported outpatient clinics, and 45 (11.4%) were unsure about admitting privileges. SCHS recognition was confirmed by 267 (67.6%). Referrals to IRs were made by 283 (71.6%), and 260 (65.8%) would increase referrals with more knowledge. The perceived benefit of additional education was as follows: no benefit (48.4%), some benefit (30.6%), and significant benefit (21.0%).

Conclusion

The study reveals gaps in awareness and knowledge of IR among family medicine doctors in Jazan. While there is recognition of IR's value and a willingness to refer patients, variations in knowledge highlight the need for targeted educational interventions. Improving education on IR could enhance integration into patient care and optimize outcomes.

## Introduction

Interventional radiology (IR) is a subspecialty of radiology that utilizes minimally invasive procedures guided by imaging technologies to diagnose and treat a wide range of conditions [1-3]. These procedures, which include techniques such as uterine fibroid embolization, coronary angiography, and vascular angioplasty, offer patients less invasive alternatives to traditional surgery, often resulting in shorter recovery times and fewer complications [4-8].

Despite the growing importance of IR in modern medical practice, awareness and understanding of these procedures among family medicine practitioners can vary significantly. Family medicine doctors, who are often the first point of contact for patients, play a crucial role in the referral process for specialized procedures. Therefore, their knowledge of and attitudes towards IR can have a substantial impact on patient care and outcomes [5,9].

In Saudi Arabia, the field of IR has seen significant advancements, with increasing recognition of its role in the healthcare system. However, the level of awareness among family medicine practitioners regarding the capabilities and benefits of IR remains underexplored. Understanding this awareness is essential for identifying gaps in knowledge and improving educational strategies to enhance collaboration between family medicine and IR.

The Saudi Commission for Health Specialties (SCHS) has recognized vascular and interventional radiology as distinct subspecialties within diagnostic radiology. Nonetheless, there is limited data on how well family medicine practitioners are informed about the scope of these subspecialties and the procedural options available. This study aims to address this knowledge gap by surveying family medicine doctors in Jazan to assess their awareness of various IR procedures and their perceived needs for further education.

By examining the current level of knowledge and the perceived benefits of increased education on IR, this study seeks to provide valuable insights that can inform the development of targeted educational programs. These programs are intended to improve the integration of IR into patient management plans and to enhance the overall quality of care provided by family medicine practitioners.

## Materials and methods

Study design

This cross-sectional study was conducted to assess the knowledge and awareness of IR among family physicians and general practitioners in the Jazan Region, Saudi Arabia. The study aimed to identify gaps in knowledge and awareness that could impact the utilization of IR services.

Study population

The study population comprised family physicians and general practitioners practicing in the Jazan region. A total of 395 participants were selected using a stratified random sampling method to ensure representation from various healthcare settings, including public and private hospitals, clinics, and health centers.

Data collection

Data were collected using an interview-administered questionnaire designed specifically for this study. The questionnaire was developed based on a review of relevant literature and expert consultations. It comprised the following three main sections:

Demographic information: This section included questions about the participants' age, gender, years of experience, and type of practice.

Knowledge of IR: This section assessed participants' understanding of various IR procedures, their indications, and their benefits. Participants were asked to respond to multiple-choice questions and case-based scenarios.

Awareness of IR services: This section evaluated participants' awareness of the availability and utilization of IR services within their region. Questions addressed familiarity with local service providers and referral processes.

The questionnaire was piloted with a sample of 15 healthcare professionals to ensure clarity and reliability. Based on the pilot test results, minor revisions were made to improve the questionnaire’s effectiveness.

Data collection process

The finalized questionnaires were distributed to the selected participants via email and in-person visits at their respective practices. A cover letter accompanied the questionnaire, explaining the study's purpose and assuring confidentiality. Participants were given two weeks to complete and return the questionnaire.

To enhance response rates, two reminder emails were sent, and follow-up phone calls were made to non-respondents. The data collection period lasted from January to March 2024.

Data analysis

Data were analyzed using Statistical Package for the Social Sciences (SPSS) Version 25 (IBM Corp., Armonk, NY). Descriptive statistics were computed to summarize demographic characteristics and responses to knowledge and awareness questions. Mean scores and standard deviations were calculated for knowledge assessments.

## Results

Demographic characteristics of respondents

The study surveyed 395 family medicine doctors in Jazan. The age distribution of respondents was as follows: 20-29 years (n=175, 44.3%), 30-39 years (n=130, 32.9%), and 40 years or older (n=90, 22.8%). Gender distribution included 176 females (44.6%) and 219 males (55.4%). Among the specialties, family medicine consultants comprised 42 respondents (10.6%), family medicine residents were 128 (32.4%), family medicine specialists were 90 (22.8%), and general practitioners were 135 (34.2%). Regarding years of practice since their last training program, 162 respondents (41.0%) had been practicing for one to two years, 137 (34.7%) for two to five years, 63 (15.9%) for six to 10 years, and 33 (8.4%) for more than 10 years (Table 1).

**Table 1:** Demographic characteristics of the respondents (n=395) Data are presented as both frequency (n) and percentage (%). Percentages may not sum to 100% due to rounding.

Characteristic		Frequency	Percent
Age in Years	20-29	175	44.3%
	30-39	130	32.9%
	40 and above	90	22.8%
Gender	Female	176	44.6%
	Male	219	55.4%
Specialty	Family medicine consultant	42	10.6%
	Family medicine specialist	90	22.8%
	Family medicine resident	128	32.4%
	General practitioner	135	34.2%
Years of practice after last training	1-2 years	162	41.0%
	2-5 years	137	34.7%
	6-10 years	63	15.9%
	More than 10 years	33	8.4%

Awareness of interventional radiology procedures

Awareness of specific IR procedures was as follows: uterine fibroid embolization (n=232, 58.7%), coronary angiography (n=227, 57.5%), vascular angioplasty for diabetic ischemic foot (n=251, 63.5%), radiofrequency ablation of tumors (n=242, 61.3%), peripheral vascular bypass procedures (n=244, 61.8%), brain biopsy (n=222, 56.2%), nephrostomy tube placement (n=235, 59.5%), treatment of varicose veins (n=249, 63.0%), and cystoscopic tumor resection (n=216, 54.7%). A proportion of respondents indicated that they did not know about these procedures or had no experience with them (Table 2).

**Table 2:** Awareness of interventional radiology procedures (n=395) Data are presented as frequency (n) and percentage (%). Percentages may not sum to 100% due to rounding.

Procedure	Don’t Know	No	Yes
Uterine fibroid embolization	65 (16.5%)	98 (24.8%)	232 (58.7%)
Coronary angiography	45 (11.4%)	123 (31.1%)	227 (57.5%)
Vascular angioplasty (diabetic ischemic foot)	58 (14.7%)	86 (21.8%)	251 (63.5%)
Radiofrequency ablation of tumors	65 (16.5%)	88 (22.3%)	242 (61.3%)
Peripheral vascular bypass procedure	74 (18.7%)	77 (19.5%)	244 (61.8%)
Brain biopsy	66 (16.7%)	107 (27.1%)	222 (56.2%)
Nephrostomy tube placement	75 (19.0%)	85 (21.5%)	235 (59.5%)
Treatment of varicose veins	60 (15.2%)	86 (21.8%)	249 (63.0%)
Cystoscopic tumor resection	82 (20.8%)	97 (24.6%)	216 (54.7%)

Knowledge and training background

Respondents' self-rated knowledge of vascular and interventional radiology was as follows: poor (n=185, 46.8%), adequate (n=107, 27.1%), good (n=62, 15.7%), and excellent (n=41, 10.4%). In terms of the training background of interventional radiologists, 248 respondents (62.8%) believed that most of their training was in radiology, 81 (20.5%) thought it was primarily in vascular surgery, and 66 (16.7%) thought it was a combination of both radiology and vascular surgery (Table 3).

**Table 3:** Knowledge and training background (n=395) Data are presented as frequency (n) and percentage (%). Percentages may not sum to 100% due to rounding.

Variable		Frequency	Percent
Self-rated knowledge of vascular and interventional radiologists	Poor	185	46.8%
	Adequate	107	27.1%
	Good	62	15.7%
	Excellent	41	10.4%
Training background of interventional radiologists	Primarily radiology	248	62.8%
	Both radiology and vascular surgery	66	16.7%
	Primarily vascular surgery	81	20.5%

Privileges and facilities of interventional radiologists

Concerning hospital privileges, 248 (62.8%) respondents reported that interventional radiologists had hospital admitting privileges, 102 (25.8%) reported they did not, and 45 (11.4%) were unsure. Regarding outpatient clinics, 251 respondents (63.5%) indicated that interventional radiologists had outpatient clinics, 89 (22.5%) believed that they did not, and 55 (13.9%) were unsure. The majority of respondents, 267 (67.6%), confirmed that the SCHS recognizes vascular and interventional radiology as a distinct subspecialty of diagnostic radiology. Of the respondents, 283 (71.6%) had referred a patient to an interventional radiologist, and 260 (65.8%) indicated that they would refer patients with more knowledge about IR. Conversely, 80 (20.3%) stated that they would not increase referrals with additional knowledge, and 55 (13.9%) were unsure. The perceived benefit of additional education about IR was reported as follows: no benefit at all (n=191, 48.4%), some benefit (n=121, 30.6%), and significant benefit (n=83, 21.0%) (Table 4).

**Table 4:** Awareness and perceived impact of interventional radiology services (n=395) Data are presented as frequency (n) and percentage (%). Percentages may not sum to 100% due to rounding. SCHS, Saudi Commission for Health Specialties; IR, interventional radiology

Variable		Frequency	Percent
Hospital privileges	Interventional radiologists have privileges	248	62.8%
	No privileges	102	25.8%
	Unsure	45	11.4%
Outpatient clinics	Interventional radiologists have clinics	251	63.5%
	No clinics	89	22.5%
	Unsure	55	13.9%
SCHS recognition of IR as subspecialty		267	67.6%
Referrals to interventional radiologists	Referrals made	283	71.6%
Increased referrals with more knowledge	Increase referrals	260	65.8%
	Do not increase referrals	80	20.3%
	Unsure	55	13.9%
Perceived benefit of additional education	No benefit	191	48.4%
	Some benefit	121	30.6%
	Significant benefit	83	21.0%

## Discussion

This study aimed to assess the awareness and understanding of IR among family medicine doctors in Jazan and to identify potential areas for improvement in educational outreach. Our findings reveal both strengths and gaps in knowledge about IR procedures among this group of healthcare providers.

Our results indicate a varied level of awareness among family medicine practitioners regarding different IR procedures. The highest awareness was observed for vascular angioplasty for diabetic ischemic foot (63.5%) and treatment of varicose veins (63.0%). In contrast, awareness was lower for cystoscopic tumor resection (54.7%) and uterine fibroid embolization (58.7%). These variations suggest that while some procedures are well-known among family doctors, others may benefit from increased visibility and educational efforts [8-10].

The disparity in awareness across procedures underscores the need for targeted educational interventions. Procedures such as vascular angioplasty and varicose vein treatment, which are associated with chronic conditions prevalent in primary care, may already be more familiar due to their direct impact on patient management. Conversely, procedures with less direct primary care relevance, such as brain biopsy or cystoscopic tumor resection, may not be as widely recognized, indicating a potential area for enhanced education [7,11].

Self-reported knowledge of vascular and IR procedures varied significantly among respondents. A substantial proportion of family medicine doctors rated their knowledge as poor (46.8%), while only a small fraction considered it excellent (10.4%). This self-assessment aligns with the observed gaps in procedural awareness and highlights a critical area for improvement [10-13].

Regarding the training background of interventional radiologists, the majority of respondents (62.8%) believed that their training was primarily in radiology, with a smaller proportion attributing it to vascular surgery (20.5%). This perception may influence how family medicine doctors understand the scope and capabilities of IR and underscores the importance of clarifying the multidisciplinary nature of IR training [8,14].

The survey results indicated that most family medicine doctors believe that interventional radiologists have hospital admitting privileges (62.8%) and outpatient clinics (63.5%). However, a notable proportion remained unsure about these aspects. The clarity on hospital privileges and outpatient facilities is crucial as it impacts the ease with which family doctors can refer patients for IR procedures.

The majority of respondents confirmed that the SCHS recognizes IR as a distinct subspecialty (67.6%). Furthermore, a significant number of family doctors reported having referred patients to an interventional radiologist (71.6%) and expressed a willingness to increase referrals with additional knowledge (65.8%). These findings suggest that while there is a general acceptance and utilization of IR services, there is also a clear need for further education to enhance referral practices and fully leverage the capabilities of IR [10-14]. The perceived benefit of additional education about IR varied, with a substantial number of respondents acknowledging some benefit (30.6%) or significant benefit (21.0%). This indicates a recognition of the value that enhanced knowledge could bring to patient care and referral practices.

Limitations

This study has several limitations that must be considered. First, the survey was conducted exclusively among family medicine doctors in Jazan, which may limit the generalizability of the findings to other regions or specialties. Additionally, the reliance on self-reported data may introduce response bias, as participants might overestimate their knowledge or awareness. The cross-sectional nature of the study also means that causal relationships between awareness, knowledge, and referral practices cannot be established. Future research should include a more diverse sample and employ longitudinal designs to better understand the impact of educational interventions on IR practices.

## Conclusions

In conclusion, this study reveals significant gaps in the awareness and understanding of IR among family medicine doctors in Jazan. While there is a general recognition of the value of IR and a willingness to enhance referral practices, substantial variations in awareness and self-reported knowledge highlight the need for targeted educational interventions. Addressing these gaps through focused educational programs could improve the integration of IR into patient care and optimize outcomes. Future research should continue to explore educational strategies and their impact on clinical practice to ensure that family medicine doctors are well-informed about the full spectrum of IR services.
